# Peep titration based on the open lung approach during one lung ventilation in thoracic surgery: a physiological study

**DOI:** 10.1186/s12871-018-0624-3

**Published:** 2018-10-31

**Authors:** Michela Rauseo, Lucia Mirabella, Salvatore Grasso, Antonella Cotoia, Savino Spadaro, Davide D’Antini, Franca Valentino, Livio Tullo, Domenico Loizzi, Francesco Sollitto, Gilda Cinnella

**Affiliations:** 1 Department of Anesthesia and Intensive care, University of Foggia, Viale Pinto, 1-71100 Foggia, Italy; 2 Department of Anesthesia and Intensive care, University of Bari, Bari, Italy; 3 Department of Anesthesia and Intensive care, University of Ferrara, Ferrara, Italy; 4 Department of Thoracic Surgery, University of Foggia, Foggia, Italy; 5 Interdepartmental Division of Critical Care Medicine, University of Toronto, Toronto, ON Canada

**Keywords:** One lung ventilation, Recruitment maneuver, Thoracic surgery, Open lung approach

## Abstract

**Background:**

During thoracic surgery in lateral decubitus, one lung ventilation (OLV) may impair respiratory mechanics and gas exchange. We tested a strategy based on an open lung approach (OLA) consisting in lung recruitment immediately followed by a decremental positive-end expiratory pressure (PEEP) titration to the best respiratory system compliance (C_RS_) and separately quantified the elastic properties of the lung and the chest wall. Our hypothesis was that this approach would improve gas exchange. Further, we were interested in documenting the impact of the OLA on partitioned respiratory system mechanics.

**Methods:**

In thirteen patients undergoing upper left lobectomy we studied lung and chest wall mechanics, transpulmonary pressure (P_L_), respiratory system and transpulmonary driving pressure (ΔP_RS_ and ΔP_L_), gas exchange and hemodynamics at two time-points (a) during OLV at zero end-expiratory pressure (OLV_pre-OLA_) and (b) after the application of the open-lung strategy (OLV_post-OLA_).

**Results:**

The external PEEP selected through the OLA was 6 ± 0.8 cmH_2_O. As compared to OLV_pre-OLA_, the PaO_2_/FiO_2_ ratio went from 205 ± 73 to 313 ± 86 (*p* = .05) and C_L_ increased from 56 ± 18 ml/cmH_2_O to 71 ± 12 ml/cmH_2_O (*p* = .0013), without changes in C_CW_.

Both ΔP_RS_ and ΔP_L_ decreased from 9.2 ± 0.4 cmH_2_O to 6.8 ± 0.6 cmH_2_O and from 8.1 ± 0.5 cmH_2_O to 5.7 ± 0.5 cmH_2_O, (p = .001 and *p* = .015 vs OLV_pre-OLA_), respectively. Hemodynamic parameters remained stable throughout the study period.

**Conclusions:**

In our patients, the OLA strategy performed during OLV improved oxygenation and increased C_L_ and had no clinically significant hemodynamic effects. Although our study was not specifically designed to study ΔP_RS_ and ΔP_L_, we observed a parallel reduction of both after the OLA.

**Trial registration:**

TRN: ClinicalTrials.gov, NCT03435523, retrospectively registered, Feb 14 2018.

## Background

During one-lung ventilation (OLV) in the lateral decubitus position for thoracic surgery, hypoxemia may occur in almost 10% of patients [1–3], due to the total collapse of the non-dependent lung and, eventually, to atelectasis in the ventilated, dependent lung [4, 5]. We recently demonstrated [6] that a recruitment strategy applied to the dependent lung followed by a moderate PEEP level results in significant alveolar recruitment, increased arterial oxygenation and reduction of the respiratory system elastance (E_RS_), without hemodynamic impairment. Our data are in agreement with reports from several physiological studies [7–10], however little is still known on how to perform recruitment and set PEEP in this context. Recent physiological data suggest that immediately after the recruitment maneuver (RM), PEEP should be titrated through a decremental trial, in order to optimize respiratory mechanics while minimizing alveolar over distension [11]. This approach, originally proposed for patients with Acute Respiratory Distress Syndrome (ARDS), is designated as the “open lung approach” (OLA). Its physiological rationale is that alveolar recruitment is a pan-inspiratory phenomenon, so that a maximal inflation is needed to reverse lung atelectasis. Furthermore, due to the hysteresis between the inspiratory and expiratory volume-pressure curve, in the OLA approach, PEEP is titrated in the expiratory limb of the respiratory system volume-pressure curve, immediately after obtaining the maximal recruitment [11, 12].

A key point in understanding the effects of the OLA strategy is the partitioning between lung and chest wall mechanical properties, through the measurement of transpulmonary pressure (P_L_), i.e. the lung distending pressure: in clinical conditions, P_L_ allows to evaluate the influence of chest wall mechanics on lung volumes and airway pressures. For a given airway opening pressure (P_AO_), pleural pressure (P_PL_) varies depending on the amount of lung compression exerted by the chest wall [13]. Therefore, when the chest wall compression rises, P_L_ drops and lungs may be underinflated, independently from the level of P_AO_ [14]. We recently demonstrated that OLA applied during laparoscopic surgery was associated with a significant increase in P_L_ and improvement in gas exchange, suggesting that PEEP and transpulmonary pressure are important determinants of respiratory mechanics [15].

The measurement of lung and chest wall response to the OLA may thus be of interest during OLV, since a multiplicity of factors may account for the alveolar derecruitment that usually develops in the dependent lung during OLV, such as the underlying lung parenchymal disease, the loss of chest wall elastic recoil, the lateral decubitus usually with a 15–20° posterior rotation of the coxa [2], the surgical manoeuvres [4], the mediastinum weight and the ventilatory set up used [16–18].

Our study was thus launched to evaluate the effect on oxygenation and on lung and chest wall mechanics of the OLA applied on the dependent lung during thoracic surgery.

## Methods

After approval of the local ethics committee and written informed consent from each patient, the study was performed in consecutive patients scheduled to undergo elective lung lobectomy or resection through thoracotomy requiring OLV in the right lateral position, from February to November 2012 (ClinicalTrials.gov: NCT03435523). Inclusion criteria were age > 18 years, surgery and OLV lasting ≥60 min. Exclusion criteria were lung reduction surgery, pneumonectomy, patients with severe COPD with preoperative forced expiratory volume in 1 s (FEV_1_) to forced vital capacity (FVC) ratio, expressed as a percentage (FEV_1_/FVC%) < 60%, presence of large bullae, pleural diseases and/or acute or chronic uncompensated cardiac disease.

Upon their arrival in the operating room, patients were pre-medicated with midazolam 0.03–0.04 mg/kg. After applying standard monitoring device [electrocardiogram and pulse oximeter (Intellivue MP40 monitor, Philips, Boeblingen, Germany)], radial artery was cannulated (Radial Artery Catheterization Set, Arrow International, Reading, PA, USA) and the cannula was connected to the FloTrac™ sensor and the Vigileo™ monitor (Edwards Life Sciences LLC, Irwine, CA, USA, software version 01.10), which, in addition to arterial pressure transduction, allowed stroke volume (SV) and stroke volume variation (SVV) estimation from the arterial pressure waveform. The level of anesthesia was assessed through bi-spectral index (BIS) monitoring (Aspect A-2000 ®; Aspect Medical System, Newton, MA). Patients were given a bolus of 8 ml/kg of normal saline IV before the induction of anesthesia and were then maintained with 5 ml/kg/h of normal saline solution. A standardized protocol for hemodynamic management was applied in order to assure fluid volume optimization [16, 17]. Briefly, if SVV was lower than 13%, no additional fluids were given whereas if SVV was higher 13%, additional boluses of 250 ml of artificial colloid were infused over 15–20 min. After each bolus SVV was re-evaluated, and a further bolus was administered if SV increased by more than 10%, until reaching a SVV lower than 13%.

Anesthesia was induced with propofol 2 mg*kg ^− 1^, fentanyl 3 γ*kg ^− 1^, and succinylcholine 1 mg*kg ^− 1^. Anesthesia was maintained with an infusion of propofol 150–200 γ*kg ^− 1^*min ^− 1^, remifentanil 0.1–0.2 γ*kg ^− 1^*min ^-1^and cisatracurium 1.5 γ*kg ^− 1^*min ^− 1^. The infusion rate of propofol was varied in order to target a BIS value between 50 and 60.

After induction, the trachea and left bronchus were intubated with a left double lumen tube (DLT) of the appropriate size (Rushelit Rush AG lab, Waiblingen, Germany). Correct DLT position was checked using fiber optic bronchoscopy and confirmed after turning the patients in the lateral position. At OLV time, the lumen of the non-ventilated lung was left open to atmosphere. The lungs were ventilated using a Fabius GS Premium (Drager, Lubeck, Germany) with a square flow waveform. Ventilation was set for a tidal volume (Vt) of 6–8 ml*kg ^− 1^ PBW, respiratory rate (RR) of 12–14 breath/min, inspiratory time (Ti) of 33% without inspiratory pause. Patients were initially ventilated using oxygen in air with an inspiratory oxygen fraction (FiO_2_) as needed to maintain the SaO2 > 95%. No PEEP was initially added. During OLV the Vt was reduced by 30% from starting value, increasing respiratory rate as needed, while all the other parameter were kept constant [6].

### Study protocol (Fig.1)

When OLV was started, after assuring that patients were hemodynamically stable (i.e. mean arterial blood pressure, mBP > 80 mmHg, HR > 60 b/min) and that the preload index continued to be adequate (i.e. SVV ≤ 13%), the recruitment manoeuver was applied selectively to the ventilated lung as already described [6, 13, 18, 19]. In summary, the ventilator was switched to pressure-control ventilation with an inspiratory pressure of 20 cmH_2_O above PEEP. After a 3 min equilibration, PEEP was applied in steps of 5,10,15 and 20 cmH_2_O every five respiratory breaths; the maximal *recruiting pressure of 40 cmH*_*2*_*O (20 PEEP/20 driving pressure) was applied for six breaths*. Subsequently, after setting an inspiratory pressure of 15 cmH_2_O above PEEP, PEEP was stepwise reduced, starting from 15 cmH_2_O, by 2 cmH_2_O every 2 min. During the decremental PEEP trial, static C_RS_ was measured at every step, through end inspiratory and end expiratory occlusions. The PEEP level corresponding to highest C_RS_ during the decremental trial was identified as the “best PEEP”. Subsequently, the lungs were recruited again and the “best” PEEP was applied. The ventilator was then switched to volume-control ventilation maintaining the baseline settings, except that for the “best” PEEP level. This setting was maintained until the end of surgery.

**Fig. 1:**
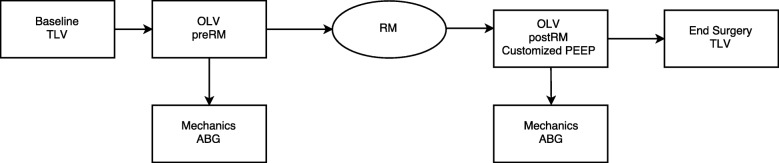
Study protocol

Hemodynamic, gas exchange and respiratory mechanics measurements were obtained in two steps, before surgery, with the chest wall closed: a) on OLV in the lateral decubitus before applying the open lung approach (OLV_pre-OLA_), and, b) 20 min after applying the open lung approach (OLV_post-OLA_).

### Measurements

Blood pressure (BP) was measured through a radial catheter connected to the pressure transducer of the MP40 monitor. Hemodynamic parameters obtained through the Vigileo included SV, cardiac output (CO), and stroke volume variation (SVV) whereas stroke volume index (SVI) and cardiac index (CI) were calculated using standard formulae. All intravascular pressure measurements were zeroed to the mid-axillary line. Analysis of arterial blood gases was performed (ABL 330; Radiometer, Copenaghen, Denmark).

Respiratory mechanics parameters were recorded, digitized and collected on a personal computer through a 12-bit analog-to digital converter board (DAQCard 700; National Instrument, Austin, TX) at a sample rate of 200 Hz (ICU Lab, KleisTEK Engineering, Bari, Italy).

Flow was measured with a heated pneumotachograph (Fleisch no.2; Fleisch, Lausanne, Switzerland), connected to a differential pressure transducer (Diff-Cap, ± 1cmH20; Special Instruments, Nordlingen, Germany) inserted between the Y-piece of the ventilator circuit and the endotracheal tube. The pneumotachograph was linear over the experimental range of flow. Volume was obtained by numerical integration of the flow signal. P_AO_ was measured proximal to the endotracheal tube with a pressure transducer (Special Instruments Digima-Clic ±100 cmH20; Nordlingen, Germany). The difference between the level of the PEEP set on the ventilator (read as the P_AO_ value at the end of a regular breath) (PEEP_external_) and the pressure in P_AO_ during a 3–5 s end-expiratory occlusion (PEEP_TOT_, _RS_) was measured and regarded as the static intrinsic PEEP of the respiratory system (PEEPi_RS_) according to Pepe [14].

P_PL_ was estimated by measurement of esophageal pressure (P_eso_), by a thin latex balloon-tipped catheter connected via a polyethylene catheter to a pressure transducer (Special Instrument DigimaClic ±100 cmH2O; Nordlingen, Germany). The esophageal balloon was filled with 1–1.5 ml of air and the correct position of the probe was verified on the computer software of recording and analysis (ICU Lab, KleisTEK Engineering, Bari, Italy) according to the literature [20–24].

The end-inspiratory and end-expiratory occlusions were performed through the inspiratory and expiratory holds on the Fabius GS Premium ventilator.

Total positive end expiratory pressure (PEEP_TOT_), P_AO_, P_PLAT_, P_L_, C_RS_, C_L_, C_CW_, ΔP_RS_ and ΔP_L_ were measured as previously described [15].

### Statistical analysis

A sample size calculation was performed using data from our previous study [6] on the effects of recruiting maneuvers on C_RS_ and PaO_2_/FiO_2_ in patients undergoing OLV for thoracic surgery. Based on these data, the significant change in C_RS_ before and after the recruitment maneuver was designated as a 25% C_RS_ increase with a SD of 8.9 and the improvement in PaO_2_/FiO_2_ as a ratio increase of 115 with a SD of 120. The larger sample size of 13 patients resulted from the P/F ratio and was used for patients’ enrollment. The α and β errors for the sample size were chosen as 0.05 and 0.90 respectively [25, 26]. Statistical comparison of respiratory mechanics, hemodynamic and gas exchange data was performed between the two study steps: data were tested for normal distribution by the Kolmogorov-Smirnov goodness- of –fit test and presented as mean (±SD). Paired T-test analysis was performed between OLV_pre-OLA_ and OLV_post-OLA_ to evaluate the effect of the recruitment maneuvers on respiratory mechanics, hemodynamic and gas exchange. A *p* value < 0.05 was considered statistically significant. Data are presented as mean ± SD. Statistical analysis was performed using the software Statistica 10.0 (Statsoft Italia srl 2011).

## Results

**Fig. 2:**
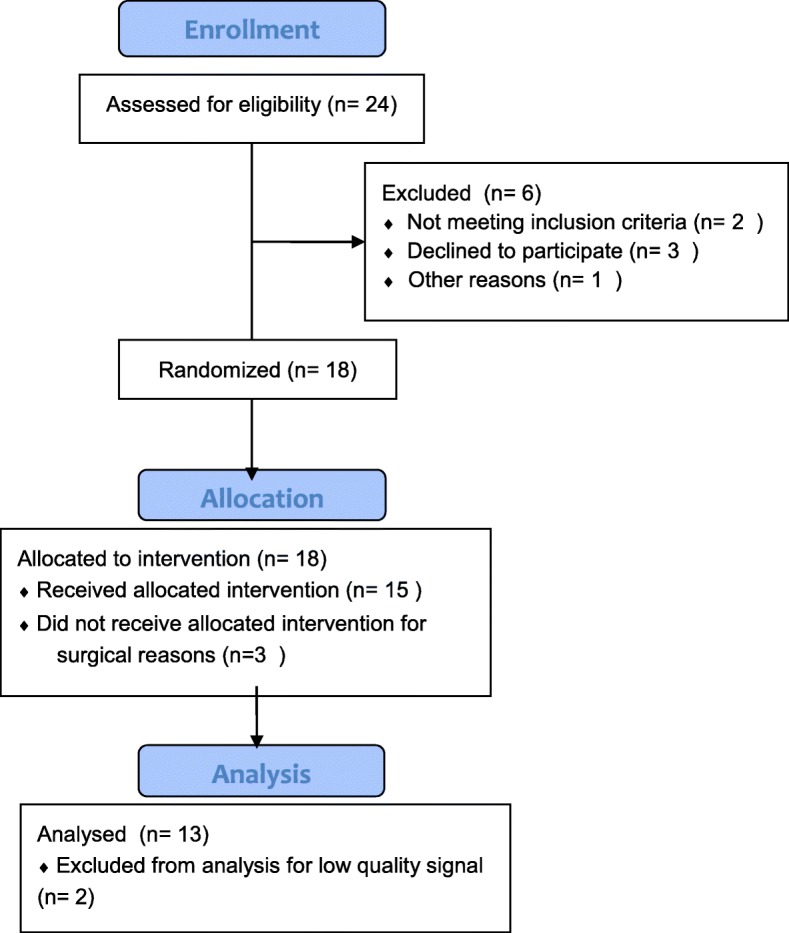
CONSORT flow chart

Thirteen out of 24 patients initially evaluated for enrolment were included in the study. The enrolment flow diagram is reported in Fig. 2. The mean external PEEP resulting from the decremental PEEP titration after the RM was 6 ± 0.8 cmH_2_O (range 5–8 cmH_2_O).

Demographic characteristics and baseline spirometric data of the population studied are presented in Table 1. The study was completed successfully in all the patients without protocol-related complications or adverse events.

**Table 1:** Patients demographic characteristics

Patient No	Age (yr)	Sex	BMI (Kg/m^2^)	ASA	pH	PaO_2_ (mmHg)	PaCO_2_ (mmHg)	FEV_1_ (% Pred)	FEV_1_/FVC (%)	Surgery	Duration (min)
1	60-69	2	21.6	2	7.31	73	53	42	67	Left upper lobectomy	75
2	50-59	2	19.64	4	7.43	76	43	55	67	Left upper lobectomy	65
3	60-70	2	20.83	3	7.42	82	38	65	77	Left upper lobectomy	80
4	50-60	2	22.18	3	7.42	108	24	87	88	Left upper lobectomy	70
5	60-70	2	25.75	3	7.42	81	48	50	60	Left upper lobectomy	70
6	50-60	2	16.86	2	7.38	73	43	115	108	Left upper lobectomy	60
7	50-60	2	22.05	2	7.42	89	47	98.2	77	Left upper lobectomy	85
8	50-60	1	20.83	3	7.4	73	42	42	67	Left upper lobectomy	90
9	40-50	2	25.75	3	7.41	89	50	69	78	Left upper lobectomy	95
10	70-80	2	22.41	2	7.38	84	38	96	99	Left upper lobectomy	105
11	60-70	2	23.33	2	7.38	113	40	57	107	Left upper lobectomy	110
12	60-70	1	19.39	4	7.4	97	43	82	71	Left upper lobectomy	85
13	50-60	2	26.36	2	7.45	63	49	108	114	Left upper lobectomy	90
Mean	59		22.07		7.4	86.4	42.6	86.6	82.3		83.1
SD	6.8		2.75		0.03	16.2	6.15	22.5	19.43		17.35

*BMI* body mass index, *FEV*_*1*_ forced expiratory volume in 1 s, *% pred* percentage of the predicted value, *FVC* forced vital capacity; FiO2 during the ABG was 21%

None of the patients was affected by postoperative pulmonary complications.

Hemodynamic parameters remained unchanged throughout the study despite a transient decrease in CI and MAP during the RM (Table 2).

**Table 2:** Hemodynamic parameters during the different experimental conditions

	TLV Baseline	OLV _preOLA_	During RM	OLV _postOLA_	TLV end	ANOVA
HR (b/m)	76 ±14	77± 12	80 ±11	76 ±13	75± 12	N.S.
MAP (mmHg)	91 ±12	86 ±15	82 ±10 *	88 ±14	90 ±11	P < 0.05
CI (l/m^2^)	2.7 ±0.4	3.1 ±0.9	2.4± 0.3 *	2.8± 0.4	3.0± 0.2	P< 0.05
SVV (%)	12± 2	13 ±1	13± 2	13± 2	14 ±1	N.S.

ANOVA *P<0.05 TLV baseline vs RM

*HR* heart rate, *MAP* mean arterial blood pressure, *CI* cardiac index, *SVV* stroke volume variation, *TLV* two-lung ventilation, *OLV* one-lung ventilation, *RM* recruitment manoeuvre. Data are expressed as mean ± SD

Table 3 shows the main respiratory mechanics and gas exchange parameters throughout the study (data are expressed as mean ± SD). On OLV_pre-OLA_ the PaO_2_/FiO_2_ ratio was 205 ± 73 and improved to 313 ± 86 (*p* = 0.05) on OLV_post-OLA_ while PaCO_2_ and pH remained unchanged.

**Table 3:** Breathing pattern and gas exchange parameters during the different experimental conditions

	OLV_pre-OLA_	OLV_post-OLA_	*p*-value
V_T_ (ml/Kg PBW)	5.9±0.9	5.9±0.7	0.47
RR (breaths/min)	18±1	18±1	0.77
P_AO, PLAT_ (cmH_2_O)	12.8±1.6	16.7±2.2*	0.003
PEEP_ext_ (cmH_2_O)	0	6±0.8	0.3
PEEP_TOT, RS_ (cmH_2_O)	3.6±1.4	9.8±1.2*	0.0001
ΔP_RS_ (cmH_2_O)	9.24±0.4	6.88±0.6*	0.001
ΔP_L_ (cmH_2_O)	8.1±0.5	5.7±0.5*	0.015
C_RS_ (ml/cmH_2_O)	45±11.3	62±13.4*	0.001
C_L_ (ml/cmH_2_O)	56.17±18.18	71.06 ±12.25*	0.0013
C_CW_ (ml/cmH_2_O)	310±15.4	312±14.2	NS
Raw (cmH_2_O/L/s)	11.24±3.72	7.42±4.21*	0.004
pH	7.37±0.05	7.37±0.03	NS
PaCO_2_ (mmHg)	44±2.9	43±3.8	NS
PaO_2_/FiO_2_(mmHg)	205±73	313±86*	0.004

Pared T-test was used for the comparison of continuous variables. Data are expressed as mean ± SD

*C*_*RS*_ respiratory system compliance, *C*_*L*_ lung compliance, *C*_*CW*_ chest wall compliance, *ΔP*_*RS*_ respiratory system driving pressure, *ΔP*_*L*_ transpulmonary driving pressure, *FiO*_*2*_ inspiratory oxygen fraction, *PaO*_*2*_ arterial oxygen tension, *PaCO*_*2*_ arterial carbon dioxide tension, *PEEP* positive end expiratory pressure, *PEEP*_*ext*_ external PEEP, *PEEP*_*TOT,RS*_ respiratory system total PEEP, *P*_*AO,PLAT*_ respiratory system plateau airways pressure, *RR* respiratory rate, *V*_*T*_ tidal volume

statistical significant when compared to OLV_pre-OLA_

Both C_RS_ and C_L_ significantly increased from 45 ± 11 ml/cmH_2_O to 62 ± 13 ml/cmH_2_O and from 56 ± 18 ml/cmH_2_O to 71 ± 12 ml/cmH_2_O (*p* = 0.001, *p* = 0.0013, vs OLV_pre-OLA_, respectively), while C_CW_ did not change.

Airway resistance (Raw) went from 11.24 ± 3.72 cmH_2_O/L/s to 7.42 ± 4.21 cmH_2_O/L/s. ΔP_RS_ and ΔP_L_ decreased after the OLA from 9.2 ± 0.4 cmH_2_O to 6.9 ± 0.6 cmH_2_O and from 8.1 ± 0.5 cmH_2_O to 5.7 ± 0.5 cmH_2_O respectively (*p* = 0.001 and *p* = 0.015 vs OLV_pre_-_OLA_).

## Discussion

The main result of the present study is that during OLV in lateral decubitus, the application of an open lung strategy consisting in a RM followed by a decremental PEEP titration significantly improves oxygenation and lung mechanical properties without significant hemodynamic effects.

It is well acknowledged that OLV in lateral decubitus may induce a “multiple-hit” mechanism for lung injury: on one side the dependent lung is prone to mechanical damage due to intratidal cyclic parenchymal opening and closing, compression by mediastinal structures, surgical manipulations and loss of elastic recoil, on the other there is the collapse and re-expansion of the non dependent lung [27]. Moreover, the reduction of aerated lung capacity may worsen the ventilation-to-perfusion matching that could result in a significant gas exchange impairment [28, 29]. The common ventilatory setting to counterbalance hypoxemia during OLV used to include Vt as high as during two-lungs ventilation (i.e. 8 to 10 ml/Kg) [7, 30–32] and PEEP as a rescue therapy in case of severe hypoxemia, in selected patients. Recent studies, however, demonstrated that large Vts do exacerbate the risk for Ventilator-Induced Lung Injury (VILI) through the enhancement of oxidative and inflammatory reactions [33], whereas a lung-protective ventilatory strategy with smaller Vt and PEEP applied after appropriate lung recruitment on the dependent lung during OLV minimizes VILI, resulting in less postoperative lung complications [34–38], and increases oxygenation [9, 18, 19, 28]. However, very low Vts as the one suggested for ventilating the dependent lung during OLV (4–5 ml/Kg) may per se induce alveolar de-recruitment unless an “adequate” PEEP level is set. Unfortunately, there is no agreement on the correct PEEP setting in the OLV context. In a recent study, Spadaro and coworkers documented the physiological interplay between very low Vts and two PEEP levels (5 and 10 cmH_2_O) on oxygenation, respiratory mechanics and ventilation/perfusion mismatch during OLV. Their findings were that in this setting only a relatively high PEEP level (10 cmH_2_O) guarantees improvement of gas exchange, shunt reduction and improvement of respiratory mechanics [39]. In the present study we found that lower PEEP levels, i.e. 6 ± 0.8 cmH_2_O (range 5–8 cmH_2_O) were able to improve oxygenation and lung mechanics. The apparent discrepancy between ours and Spadaro’s data could be explained by the different design of the two studies. Indeed, we applied a complete lung recruitment and individualized PEEP titration strategy whereas Spadaro studied two fix PEEP levels were applied without previous maximal lung recruitment. Our study seems to confirm that in the OLV context, similarly than in early ARDS, a decremental PEEP titration trial is required to keep the lung fully opened after a maximal lung recruitment maneuver [40].

In a previous report, our group demonstrated that, besides the positive effect on gas exchange, the application of a RM followed by a standard PEEP of 5 mmHg during OLV induces a reduction in E_RS_ [6]. Recently, Ferrando et al. [11], showed that the improvement in C_RS_, dead space and oxygenation during OLV was more noticeable in patients undergoing a PEEP titration after the RM. Actually, individualizing the level of PEEP required to maintain the lung open after the RM, is presently considered superior to the “one-size-fit-all” method of standardized PEEP in order to match that particular patients respiratory mechanical properties [41]. Currently, the appropriate method to select PEEP remains unclear, however the decremental PEEP titration method that we used in the present study and that lead to an average PEEP level of 6 ± 0.8 cmH_2_O, is widely studied and relatively easy to perform at the bedside, even during anesthesia [11, 18, 19]. When compared to Ferrando et al. [11], we used the same recruitment maneuver and observed an improvement in gas exchange and C_RS_. The novelty of our study is that we partitioned lung and chest wall mechanics response to the OLA strategy, which allowed to highlight how this response is attributable mainly to the lung component. In literature, an amount of data confirm that the dependent hemithorax is compressed in lateral position: actually, the static P/V curve of the lungs has a rightward horizontal shift in lateral decubitus as compared to the upright position (i.e. about15% less compliant), with a similar slope [42]. By separately measuring the C_RS_ of both hemithoraces, Klingsted et al. [43], in a seminal paper demonstrated that the C_RS_ of the dependent hemithorax is about two-thirds of the contra lateral one when both lungs are ventilated in lateral decubitus (as during differential lung ventilation (DLV)). Interestingly, they also found that when switching to OLV, the static airway P/V curve of the dependent lung (super-syringe method with inflation of 1.5–2 l), is shifted to the left and steeper, as compared to the P/V curve obtained in the same lung during DLV, and therefore demonstrated a greater potential for lung recruitment with the application of selective PEEP. The same was observed for the esophageal P/V curve, which was shifted to the left and steeper, showing a small increase in pressure for the whole volume range. This suggests that during OLV the elastic recoil of the non-dependent, collapsed lung and the “compression” that it may exert on the dependent lung are lower than the elastic, expanding force in the chest wall. Our results seem to confirm these data. We measured respiratory mechanics on the dependent lung and found that on OLV_pre_, the chest wall contributed roughly by 30% to total respiratory system elastance whereas the lung contributed by 70%. After implementing the OLA strategy, the absolute chest wall elastance did not change and therefore the decrease in total respiratory system elastance was due essentially to the decrease in lung elastance.

The reduction in inspiratory resistances observed, further suggests a more even distribution of Vt within the lung following the opening up of collapsed alveoli. Moreover, it seems reasonable to hypothesize that patients lateral position per se (surgical bed tilted in mild reverse Trendelenburg to allow a better surgical exposure, and consequent reduced abdominal content’s backpressure against the diaphragm [38]), keeps chest wall mechanics invariable, as demonstrated by the unchanged E_CW_ absolute average value after the OLA.

An additional result of the present study is the reduction of both airways and transpulmonary driving pressure following the OLA strategy. Our study protocol was not specifically designed to study driving pressures: PEEP titration was indeed performed in order to obtain the highest C_RS_ and not the lowest ΔP. In that case, actually, different PEEP level would probably have been reached, anyway we believe that this unanticipated result does deserve some considerations.

An increasing amount of data in literature suggests that driving pressure is a predictor of mortality in ARDS patients [44, 45], and a key mediator of the effects of various ventilatory interventions [5, 6, 15]. ΔP is the tidal change in static trans-respiratory pressure, measured as P_PlatRS_-PEEP_tot_, in response to a volume-controlled breath and expresses the ratio between Vt and respiratory system compliance Vt/C_RS_ [45]. Many data show that reducing the alveolar cyclic collapse and reopening (atelectrauma) may protect lungs from VILI [44, 45]. Therefore, a ventilatory setting aimed at obtaining the lowest ΔP is suggested as method for personalizing ventilation to individual lung physiology. The reduction in ΔP following recruitment manoeuvres and/or PEEP application is also regarded as a realistic target for ventilatory adjustments. Moreover, measuring the tidal change of the real lung-distending pressure, i.e. ΔP_L_, is suggested as a useful tool to understand how much of the ΔP is due to the lungs and how much to chest wall elastance [45]. The relationship between the two driving pressures is not thoroughly clarified; however, in our patients the parallel reduction of both driving pressures measured during volume-controlled ventilation after the OLA as compared to the ones measured before the OLA, may suggests its efficacy in terms of alveolar recruitment and, if confirmed by further studies, could corroborate the usefulness of ΔP monitoring.

We also confirm that a correct hemodynamic management during the OLA strategy, does allow a safe maneuver: indeed, once the volemic status was optimized through the SVV, the OLA did not affect hemodynamic, if one excludes transient and not clinically relevant decrease in CI and PAM during the RM.

This study has nonetheless a few limitations. Firstly, we could not measure recruited volume, since we needed to keep respiratory mechanics measurement as short as possible in order not to prolong surgery. As regards esophageal pressure measurement, its pro and cons as a surrogate for transpulmonary pressure have been widely debated in literature [21–24], and any further discussion is beyond the scope of the present study. Nonetheless, the feasibility of P_es_ and the signal quality could be questioned when switching from supine to lateral position, since the heart, the mediastinum, and the weight of the surrounding parenchyma could compress and modify the balloon’s shape. However, we believe that these shortcomings should have been counterbalanced by having P_es_ waveforms always collected by the same operator (MR), and the Baydur test and the filling volume test repeatedly performed within the duration of the surgery.

Although the sample population size was small, as is the rule for physiologically oriented cross over studies like ours, our data suggest that the OLA strategy could optimize oxygenation and lung mechanics during OLV. However, physiological improvements not necessarily translate in improvements in clinically meaningful outcome parameters. Recently, a large RCT in patients undergoing major abdominal surgery was not able to show any advantage of a perioperative OLA strategy over standard treatment in terms of a composite endpoint of postoperative pulmonary and systemic complications [46]. Nevertheless, due to the peculiarities of OLV, the potential impact of OLA during OLV in a large RCT is worth to investigate and the ongoing PROTHOR study (will hopefully add more definitive knowledge in this field (clinicaltrials.gov ID NCT02963025).

## Conclusions

The results of our study provide evidence that decremental PEEP titration on the best C_RS_, immediately after maximal lung recruitment, improves oxygenation and lung mechanics. Beyond these clear physiological benefits, the eventual impact of the OLA strategy on patients outcome is yet unknown and warrants further clinical studies.

## Abbreviations

% pred: percentage of the predicted value
ARDS: Acute Respiratory Distress Syndrome
BIS: Bi-spectral index
BMI: Body mass index
BP: Blood pressure
CI: Cardiac index
C_L_: Lung compliance
CO: Cardiac output
C_RS_: Respiratory system compliance
FEV_1_: Forced expiratory volume in 1 s
FiO_2_: Inspiratory oxygen fraction
FVC: Forced vital capacity
HR: Heart rate
mBP: Mean arterial blood pressure
OLA: Open lung approach
OLV: One lung ventilation
PaCO_2_: arterial carbon dioxide tension
P_AO,PLAT_: Respiratory system plateau airways pressure
PaO_2_: arterial oxygen tension
PEEP: Positive end expiratory pressure
P_eso_: esophageal pressure
P_L_: Transpulmonary pressure
RM: Recruitment maneuvers
RR: Respiratory rate
SV: Stroke volume
SVV: Stroke volume variation
VILI: Ventilator induced lung injury
V_T_: Tidal volume
ΔP_RS_ and ΔP_L_: Respiratory system and transpulmonary driving pressure
